# Treatment-Resistant Mood Disorders

**DOI:** 10.12669/pjms.40.9.8245

**Published:** 2024-10

**Authors:** Nadia Naeem, Rabia Farooqi

**Affiliations:** 1 Nadia Naeem, Department of Psychology, University of Central Punjab, Lahore, Pakistan; 2 Rabia Farooqi, PhD. Associate Professor, Department of Psychology, University of Central Punjab, Lahore, Pakistan

**Keywords:** Treatment-resistant mood disorders, Clinical features, Evidence-based psychotherapeutic interventions, Evidence-based non-medical treatment approaches for TRMD

## Abstract

**Objective:**

The objective of the study was to explore the presentation of symptoms in patients suffering from treatment-resistant mood disorder (TRMD), and associated clinical features, including age, gender, and comorbid conditions, that lead towards the development of treatment-resistant mood disorder. Furthermore, the study analyses the available psychotherapeutic treatment modalities and evidence-based non-medical treatment approaches for TRMD.

**Method:**

The current study utilized a systematic review approach where 37 articles were studied and three articles were theoretically sampled to authenticate and signify the findings. PRISMA guidelines were followed and the review was conducted by searching social sciences databases and electronic libraries including Google Scholar, Sage Journal, and Science Direct (1990-2020).

**Results:**

The results suggested that psychotherapeutic interventions including cognitive behavior therapy, mindful-based cognitive therapy; and interpersonal and social rhythm therapeutic interventions are efficacious modalities for treatment when in augmentation with psychopharmacological treatment. Bipolar diathesis and comorbid conditions of anxiety and personality disorders are possible causal factors in developing the condition of treatment resistance in mood disorders. The prevalence rate of TRMD is more common in females and in late adulthood. The analysis also suggests that there are numerous risk factors contributing to making a mood disorder treatment-resistant over a period of time; the most observed conditions were physical and psychological comorbidity, inaccurate diagnosis, lack of proper medical treatment, illness severity, and late age diagnosis. In evidence-based non-medical treatment approaches aerobics demonstrated promising results in improving the condition of complex mood disorders.

**Conclusion:**

It was concluded that psychotherapeutic interventions in augmentation with pharmacological modalities enhance the efficacy of treatment.

## INTRODUCTION

The world has been plagued with many diseases over time and has posed great threats and challenges for the health sector. Inevitably, this has been a continuous cycle of human growth. However, what develops this concern into a more potent threat is a failure of effective treatment. There has been continuous development in the field of pharmacokinetics but many phenomena remain unknown and need to be further explored. The best possible solution is to utilize all the available resources and data for the invention of multidimensional strategies in the hope of an effective treatment outcome.

The study has explored the multiplicity of defining terms on resistance towards treatment in patients suffering from mood disorders. It is aimed that all the possible facets are explored that can contribute to a fair prognosis. It is foremost important to understand the underlying mechanism before suggesting and devising a suitable plan of action. Research gaps must be identified for successful exploration of the phenomenon. Treatment resistance cannot be comprehended exclusively by defining criteria. Therefore, a comprehensive clinical picture must be delineated involving significant aspects regarding the development of illness, related facets (age, gender, comorbid conditions) and possible treatment options which helps in critically evaluating treatment plans.

Treatment-resistant depression (TRD) refers to two adequate trials of antidepressant’s (AD’s) from different classes with different presumed mechanisms of action^1^. Treatment-resistant mania (TRM) is the maximal tolerated lithium in combination with valproate or carbamazepine for a period of three times the average cycle length, or six months, whichever is longer, in the absence of antidepressants or other cycle-promoting agents.^2^ The course of treatment-resistant mood disorder (TRMD) is a continuum ranging from partial response to complete treatment resistance rather than an all-or-nothing phenomenon. Eighty percent of patients who required multiple AD trials relapsed within the first year following remission (Readmission & Premature Death): STAR*D studies.^3^ Considerable features that should be kept in mind include pseudo resistance including misdiagnosis and inadequate treatment such as compliance, dosage, duration, and AD’s trials.^4^ Other factors might involve age, having early onset before eighteen years and in some cases late onset after sixty years.^5^

Gender is another factor as women demonstrate less responsiveness to AD’s trials.^6^ Substance Use Disorder, Anxiety Disorders, Post-Traumatic Stress Disorder (PTSD), Panic Disorder, Obsessive Compulsive Disorder, and Poor Social Adjustment are the most common comorbid conditions.^7^-^9^ Evidence-based practices include pharmacotherapy, augmentation including cognitive behavior therapy and interpersonal therapy. There is increasing awareness that the majority of depressed patients either fail to respond to an appropriate antidepressant drug trial or present a partial response, with substantial residual symptomatology and, as a consequence, an increased risk of relapse.^10^ Several pharmacological strategies have been developed for depressed patients who fail to respond to standard drug treatment,^5^,^10^-^13^ but limited research has been done on non-pharmacological approaches for TRD. The basic clinical questions are when and for whom psychotherapy should become a treatment option. In TRD, there is a need for augmenting practice guidelines with patient-specific recommendations that take into account individual variables such as history and previous treatment response.

When a psychotherapeutic intervention is planned in the setting of current drug treatment, the choice of switching or augmenting strategies should be guided by clinical judgment. When switching is endorsed, it is generally wise to postpone it to a later phase of psychotherapy, also because discontinuation symptoms, that do not necessarily abate in a couple of weeks, may have an unfavorable impact on the initial phase of psychotherapy.^14^ The study in hand focused on identifying the functional consequences of treatment resistance among mood disorders, its manifestation and trajectory, and important clinical features to understand the available evidence-based treatments in a better way.

## METHODS

### The Review Question:

- What is the presentation of treatment-resistance in mood disorders?
- What are the clinical features (age, gender, comorbid conditions) associated with TRMD?
- What are the probable factors leading to treatment resistance among mood disorders?
- What are available evidence-based psychotherapeutic interventions for TRMD?
- Which is the most successful psychotherapeutic approach for TRMD?
- What is the evidence-based non-medical treatment approaches for TRMD?

The systematic review was conducted through searching social sciences databases and electronic libraries including Google Scholar, Sage Journal and Science Direct (1990-2020). The keywords that were searched included treatment-resistant mood disorders (TRMD), development of TRMD, clinical features of TRMD, age onset of TRMD, gender differences in TRMD, and comorbid conditions of TRMD, evidence-based psychotherapeutic intervention for TRMD, evidence-based non-medical treatment approaches for TRMD. In addition, the citation of reviewed articles was further explored.

The searched results were studied including titles, abstracts, and the full copy, and screened to ensure the parameters of inclusion and exclusion criteria as follows:

- All the studies must include a psychotherapeutic intervention.
- All the studies included defined TRMD based on the criteria of resistance to medication.
- Depression was studied separately, rather than as a component of Bipolar Disorder and/or Major Depressive Disorder.
- The outcome was comprehensibly and statistically reported in experimental studies.
- Bipolar depression was separately reported apart from Unipolar Depression.
- Experimental studies had a control group.

After the screening process, a quality assessment was carried out to eliminate potential biases including culture bias, time lag bias, and outcome reporting bias. A total of forty articles were included in the current systematic review.

## RESULTS

The data search by keywords yielded seventy articles and forty articles met the inclusion criteria; three of which were theoretically sampled to match the findings. These studies were categorized into review papers, systematic reviews and meta-analyses, descriptive and exploratory researches, and experimental design researches including socio-demographic variables, clinical features, and definition of treatment resistance in mood disorders and outcome of psychosocial interventions; Google Scholar (25%), Science Direct (35%), Taylor and Francis (5%), Springer Link (7.5%), Sage Journal (7.5%), Willey Online Library (10%), National Library of Medicine (5%), and Cambridge University Press (5%).

## DISCUSSION

Comprehensive analysis of the literature revealed the course of illness in treatment-resistant depression (TRD) is expectedly worse: severity is higher, relapse is more frequent, and patients experience greater functional impairment compared to those with uncomplicated MDD.^3^,^15^,^16^ One of the most significant findings of the study was that treatment-resistant disorders can be treated more efficaciously when in augmentation with psychotherapeutic interventions. Utilizing new drug trials have been found to be rather ineffective. The efficacy of psychotherapy for treatment resistance in depression using a meta-analysis along with meta-regression was studied, the results demonstrated that augmenting psychotherapy with usual routine treatments of drug therapy and neurostimulatory treatments for the condition of TRD is well justified and leads to better and productive results as compared to pharmacological treatment alone.^17^

Findings of experimental research illustrated that the most powerful tool for psychotherapeutic intervention lies within cognitive behavior therapy. Treatment resistance in mood disorders may include non-conclusive or wrong diagnosis that is an expression of the inability to manage bipolar disorder, a manifestation of psychotic or melancholic depression, misdiagnosed non-melancholic conditions, secondary depression, and organic determinants. Studies have demonstrated that clinical features were consistent across existing literature, particularly late age and female gender were the predominant demographics affecting the trajectory of TRMD.

Findings of many studies have illustrated that bipolar disorder and co-morbid conditions including anxiety disorders and personality disorders specifically have a significant relationship with the category of mood disorder. Furthermore, studies have stated this as a term of “bipolar diathesis” referring to bipolar disorders as the beginning or the result of a TRD or mood disorder. One culture-specific study reflected that obesity is also linked with TRMD based on the prevalence of the demographics in a clinical sample. They explored the bidirectional relationship of body mass index and its treatment outcome with treatment-resistant depression among adolescents. The results of the study established that being in the overweight category did not affect the overall response toward treatment and otherwise i.e. the efficacious treatment for depression did not affect the weight or body mass index (BMI).^18^

**Figure:**
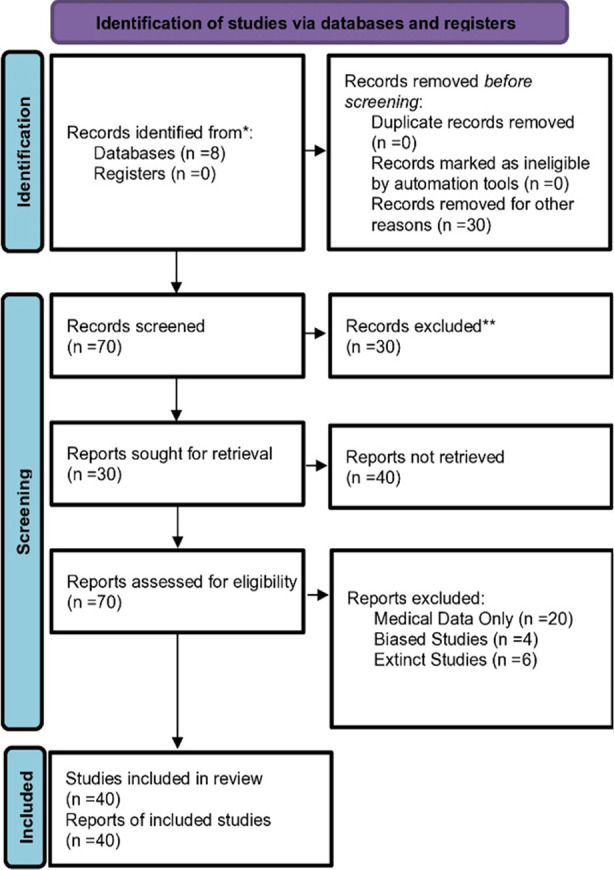
Flow Chart.1.

**Figure:**
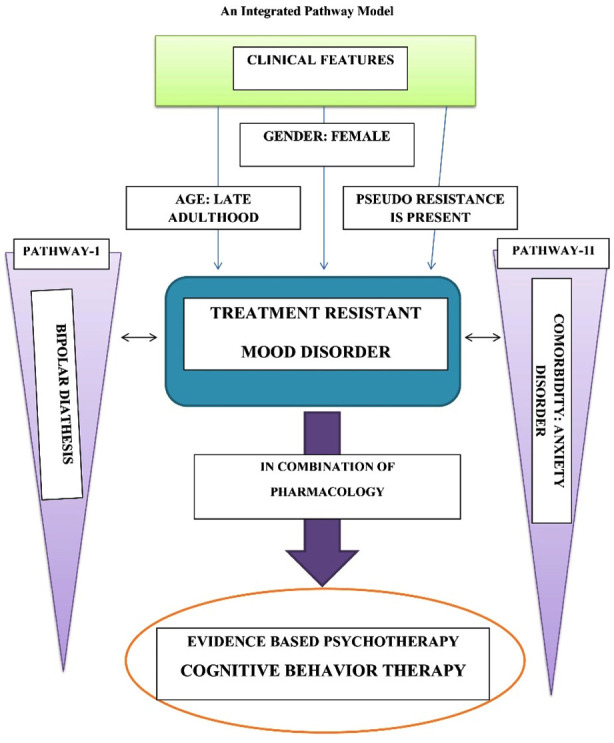
Flow Chart.2.

**Table-I:** Description of studies included.

Author	Year	Research Design	Duration of the study	Conclusion
Kornstein & Schneider	2001	Review Article	Not specified	Treatment resistance may be pseudo in nature positing the cause of inadequate drug trials, existence of secondary mood disorder or depressive subtype. Risk factors include comorbid medical and psychological conditions, older age and illness severity.
Thase et al.	2001	Review Article	Not specified	TRD is a diverse condition. Poor therapeutic alliance, low social care, life stressors, prolonged misfortune, personality traits including neuroticism or pessimism. Therapeutic approaches focused on depression are more valuable.
Nemeroff	2007	Review article including	Experimental study conducted in 2005	The prevalence rate TRD stage-I is 12 months and 3% and 2% for TRD Stage-II. It is a neglected health issue which fails in accurate diagnosis.
Vieta & Colom	2011	Review article	Not specified	CBT was found to be effective for TRD but efficacy for treatment-resistant bipolar disorder is yet to be established.
Shelton et al.	2010	Review article	Not specified	TRD cannot be defined solely by only one, predictable pathophysiological process. Various contributory factors may affect the degree of resistance to treatment and probability of response towards different psychotherapeutic interventions.
Wijeratne & Sachdev	2008	Systematic Review	1995-2007	Client’s response to treatment has been linked with many sociodemographic factors along with a client’s belief regarding depression and treatment preferences.
Rizvi et al.	2014	Experimental Study	October 2008 & August 2009	Higher rates of disability and comorbidity to other disorders (Axis 1-3) were associated with resistance to treatment, and one of the most common facets was being overweight.
Lee & Dunner	2008	Systematic review of experimental studies	January 2004 & September 2005	Early onset, comorbid conditions of anxiety disorder and substance use disorder, and higher rates of suicidal attempts are common correlates in TRMD. In women, anxiety condition has higher comorbidity.
Kenny & Williams	2007	Clinical Audit	2004	The efficacy of MBCT was studied in depressed patients in a longitudinal study suggesting improvement in depression with an effect size of 1.04. Furthermore, the participants experienced remission with some returning to experiencing normal or near to normal levels of mood.
Sharma et al.	2005	Chart review of outpatient clients	June 2000 – May 2001	The 80% of patients diagnosed with bipolar disorder manifested evident symptoms of bipolarity and were referred to as depressive patients. A frequent treatment option was a switch towards mood stabilizers. Findings posit that unipolar depression converts into TRMD due to bipolar diathesis as no response was shown on the drug trial of antidepressants.
Parker et al.	2005	Descriptive Research	2002- 2004	Failure to diagnose or misdiagnosis leading to inability towards managing bipolar disorder, depression with psychosis, melancholic depression, misdiagnosis of non-melancholic conditions, secondary depression, and organic determinants leads to TRMD
Correa et al.	2010	Systematic Review	January 1998 & January 2008	Bipolar disorder (BD) is a contributory factor towards developing TRD. Important to analyse soft signs which can modify diagnosis and treatment strategies.
McIntyre et al.	2014	Systematic Review	1980 - April 2013	Use of physical exercise specifically aerobics in the presence of social support and use of some psycho-stimulants to counter treatment resistance in depression. CBT was also found effective for TRD.
Voineskos et al.	2020	Systematic Review	Not specified	Understanding its pathophysiology, response pattern, and difference of manifestation from MDD, so that effective diagnosis leads to effective treatment saves patients time, frustration and hopelessness.
Al-Harbi	2012	Systematic Review	1990–2011	Half of the patients suffering from TRD demonstrate good response when psychotherapeutic interventions are used in augmentation with psychopharmacology; more specifically towards CBT when adjunct with mindfulness.
Souery et al.	1999	Review Article	1997	Many variables are interlinked co-interacting with TRMD not primarily linked with patient characteristics such as misdiagnosis and inadequate treatment.
Gitlin	2006	Feature Review	24 January, 2006	It was found that when substantial attention is given to psychoeducation and enhancement of coping strategies in clients, treatment resistance can be countered.
Van Bronswijk, et al.	2019	Meta-analysis	Publications up until and including 19 December, 2016 were reviewed	Augmentation to psychotherapeutic intervention is quite useful and is justified for depressive patients suffering from resistance towards treatment as compared to pharmacological and neurostimulatory treatments alone.
Keating, et al.	2018	Observational Research	April 2012 to July 2015	Physical exercise, aerobics in particular is effective when combined with social support among both youth and adults suffering from complex mood disorders.
Mansoor, et al.	2013	Experimental Study Design	2009- 2010	No relation was found among treatment resistance in depression and obesity.
Fountoulakis, et al.	2020	Systematic Review	2018	Psychoeducation is preferentially efficacious in patients with less than 7 episodes, while it has no effect in those with more than 14 episodes.
Fornaro, et al.	2020	Systematic review and exploratory meta-analysis	May 25^th^, 2020	TRMD is associated with high rates of morbidity, suicide attempts, polypharmacy, as well as extensive use of healthcare services and hospitalization
Parker & Graham	2017	Experimental study design	2008- 2013	Late-onset, female gender, older age, family history of depression, unemployment, lifetime stressors, medical conditions, comorbidity with anxiety and personality disorders, and higher use of benzodiazepines allied with treatment resistance in bipolar disorder.
Poon, et al.	2012	Systematic Review	February, 2012	Effective pharmacological treatment for treatment resistance to depressive episode in bipolar disorder involves the use of antipsychotics, NMDA, dopamine agonists, calcium-channel blockers, and thyroid hormones. Effective psychotherapeutic interventions include behavioral therapy. Other techniques include sleep deprivation, light therapy, electroconvulsive therapy, transcranial magnetic stimulation, and deep brain stimulation.
Kessler, et al.	2013	Comparative Analysis	January, 2013	Neurocognitive impairment is more common in bipolar disorder I as compared to bipolar disorder II. The most common feature was processing speed.
Machado-Vieira & Soares	2007	Literature Review	Not Specified	Review highlights genetic role, family history, lack of medical adherence, misdiagnosis, use of inappropriate pharmacological approaches, life and personal stressors with dysfunctional mood are accountable for TRMD.
Salloum & Thase	2002	Literature Review	Not Specified	Comorbid conditions such as substance use disorder specifically alcohol abuse are leading factors in causing resistance towards treatment while complicating the course and prognosis of bipolar disorder.
Hidalgo-Mazzei, et al.	2018	Survey Method	March, 2018	Clinically defined criteria for resistance towards treatment in bipolar disorder is a failure to achieve the state of remission during consecutive eight symptomatic weeks after two different treatment trials, at adequate therapeutic doses, having at least two recommended mono-therapy treatments or at least one mono-therapy treatment and another combination treatment in augmentation.
Fava	2003	Exploratory Research	2002	Certain forms of time-limited psychotherapy, such as CBT and interpersonal therapy, are efficacious in the treatment of MDD, one can argue that our current view of what constitutes an adequate antidepressant therapy is somewhat narrow and pharmaco-centric.
Maalouf, et al.	2011	Review Article	January 1980 to October 2010	Lack of medical adherence and compliance to the treatment, misdiagnosed condition; comorbid conditions (physical and psychological), life and personal stressors can correlate to TRMD. In case of lack of response to a first SSRI trial directs towards switching to another SSRI and addition of CBT and if this step fails or is suboptimal, next reasonable steps include augmentation and switching options that are evidence-based in adults.
Isai, et al.	2020	Longitudinal Study Design	18^th^ September, 2012	Treatment in combination with psychotherapeutic interventions is producing effective results, group therapy is efficient with short duration producing noticeable improvement among people suffering from treatment-resistant bipolar disorder.
Dudek, et al.	2010	Retrospective Study	December, 2009	Resistance to treatment in patients having major depression is linked with early onset of a disorder, higher rate of episodes, and inability to achieve remission.
Eisendrath, et al.	2011	Case Study	Not Specified	Mindfulness-based cognitive therapy helps prevent relapse through mindfulness meditation, decreasing rumination, enhancing self-compassion, increasing acceptance, and decreasing avoidance among patients suffering from refractory depression.
Joyce, et al.	2016	Literature Review	October, 2015	Early approaches to treatment in bipolar disorder with a staging model of illness provide evidence to improve patient outcomes as compared to otherwise.
Pandarakalam	2018	Review Article	Not Specified	Using pharmacotherapy in the acute phase and CBT in the residual phase is likely to reduce the rate of relapse in TRD.
Souza, et al.	2016	Experimental Research Design	Not Specified	Pharmacotherapy along with clinical care was effective in alleviating depressive symptoms; however, interpersonal therapy was not particularly useful when used in augmentation for depressive symptoms.
Haukson, et al.	2017	Experimental Research Design	Not Specified	CBT helped in improving the severity level of experienced symptomologies after treatments leading to an eighteen-month follow-up when used in combination.
Isasi, etal.	2010	Experimental Research Design	2005 to 2006	Augmentation with psychotherapeutic interventions is a better strategy as it reduces hospitalization rates, symptoms of depression and anxiousness were reduced over six months and adaptability to everyday life was enhanced at a termination phase.
Wells, et al.	2012	Experimental Research Design	Not Specified	Mindfulness allied with cognitive therapy showed statistically significant improvements in depression symptoms, rumination and metacognition even when used for a brief duration which were consistent over a six and later twelve-month follow-up.
Wiles, et al.	2016	Longitudinal Study Design	Nov 4^th^, 2008 to Sep 30^th^, 2010	Using CBT in combination showed longitudinal results in patients with refractory depression. Improvement in quality of life was also reported over an average of forty-six months; forty months after the end of therapy.

Moreover, an experimental study finding illustrated that along with psychotherapeutic interventions, aerobics is also an effective exercise to counter TRD. They carried out research to study the effects of physical exercise on complex mood disorders in a twelve-week program among youth and adults. The results found that when aerobics is practiced in a supportive group setting, mood symptoms improve over time given that social support is also being received which was perceived as an important factor in the program’s success.^19^ However, it was further proposed that the research is required to identify specifically the mechanisms underlying the therapeutic benefits associated with therapy programs utilizing physical exercises.

## CONCLUSION

The findings of the review illustrate those psychotherapeutic interventions, such as cognitive behavior therapy, mindful-based cognitive therapy and interpersonal and social rhythm therapeutic interventions are effective treatment modalities when used in augmentation with psychopharmacological treatment. Furthermore, it was found that when substantial attention is given to psychoeducation and enhancement of coping strategies in clients, treatment resistance can be countered. Bipolar diathesis and comorbid anxiety and personality disorders are possible clinical features associated with treatment resistance in mood disorders.

Treatment resistance in mood disorders may include a lack of appropriate diagnosis which will result in an inability to manage bipolar disorder, depression with psychosis or melancholic depression. Misdiagnosis of non-melancholic conditions, secondary depression, and organic determinants can also be a related causal factor. Development of mood disorders is more common in late age and female gender. Obesity is found to have no link with treatment resistance in mood disorders. Aerobics have been found to be a possible non-medical treatment approach in improving the condition of complex mood disorders when used in combination with psychotherapy and social support.

### Authors Contribution:

**NN:** Initiated the literature review, and analysis, synthesized the initial draft of a manuscript, and revised the draft.

**RF:** Conceptualized the idea, conceived the review, guided analysis, and critically revised the manuscript.
